# A Prospective, Randomized, Masked, and Placebo-Controlled Efficacy Study of Intraarticular Allogeneic Adipose Stem Cells for the Treatment of Osteoarthritis in Dogs

**DOI:** 10.3389/fvets.2016.00081

**Published:** 2016-09-16

**Authors:** Robert Harman, Kim Carlson, Jamie Gaynor, Scott Gustafson, Sarit Dhupa, Keith Clement, Michael Hoelzler, Tim McCarthy, Pamela Schwartz, Cheryl Adams

**Affiliations:** ^1^VetStem Biopharma, Inc., Poway, CA, USA; ^2^VCA Bay Area Veterinary Specialists, San Leandro, CA, USA; ^3^Peak Performance Veterinary Group, Frisco, CO, USA; ^4^Surgery 4 Pets, Portland, OR, USA; ^5^California Veterinary Specialists, Carlsbad, CA, USA; ^6^Burnt Hills Veterinary Hospital, Burnt Hills, NY, USA; ^7^Garden State Veterinary Specialists, Tinton Falls, NJ, USA; ^8^Cascade Veterinary Referral Center, Tigard, OR, USA; ^9^Animal Medical Center of New York, New York, NY, USA; ^10^Integrative Sports Rehab and Wellness LLC, Foxfield, CO, USA

**Keywords:** adult stem cells, clinical translation, dog model, mesenchymal stem cells, arthritis, adipose stem cells

## Abstract

Osteoarthritis (OA) is a degenerative joint disease with a high prevalence in dogs. Mesenchymal stem cells (MSCs) have been used to treat humans, dogs, and horses with OA. This report describes a prospective, randomized, blinded, and placebo-controlled clinical efficacy study of intraarticular allogeneic adipose stem cells for the treatment of dogs with OA. Health assessments and measurements of pain and activity impairment were performed at baseline and at selected time points through day 60. The primary outcome variable was the owner Client-Specific Outcome Measurement (CSOM) and secondary measures included veterinary pain on manipulation, veterinary global score, and owner global score. The dogs were treated with either a saline placebo or a single dose of allogeneic adipose-derived MSCs in either one or two joints. Seventy-four dogs were statistically analyzed for efficacy outcomes. Success in the primary outcome variable, CSOM, was statistically improved in the treated dogs compared to the placebo dogs (79.2 versus 55.4%, *p* = 0.029). The veterinary pain on manipulation score (92.8 versus 50.2%, *p* = 0.017) and the veterinary global score (86.9 versus 30.8%, *p* = 0.009) were both statistically improved in treated dogs compared to placebo. There was no detected significant difference between treated and placebo dogs in the incidence of adverse events or negative health findings. Allogeneic adipose-derived stem cell treatment was shown to be efficacious compared to placebo. This large study of dogs also provides valuable animal clinical safety and efficacy outcome data to our colleagues developing human stem cell therapy.

## Introduction

Canine osteoarthritis (OA) is a degenerative disease of all joint tissues, results in loss of articular cartilage, and has the hallmark clinical sign of pain (1). The prevalence of OA in dogs in the United States is estimated to be 20% (2). Although the most clinically obvious pathologic changes in the joint are in the articular cartilage, a closer evaluation shows that the pathology encompasses the entire joint, including the synovium, tendons, ligaments, bone, and neural tissues (1). The pathophysiological mechanisms of OA have been well described (2, 3), including the key role that inflammatory and regulatory cytokines play in homeostasis and degradation (3). Traditional multi-modal therapy is based primarily on reducing inflammation and pain; this typically includes long-term cyclo-oxygenase-inhibiting non-steroidal anti-inflammatory drug (NSAID) therapy, physical therapy, diet and weight management, and dietary supplements (1). Although NSAIDs are typically prescribed for a long duration, average owner compliance with prescriptions for daily NSAID administration may be poor (4), which leaves dogs with untreated clinical signs. There is a need for products with a profile of convenient extended relief of pain. Cell therapy relieves the owner of the burden of daily administration, may be associated with tissue regeneration, and should be investigated as a therapeutic solution. Autologous cell therapy is commonly used in veterinary practice, but the need for a surgical collection limits its use. Allogeneic stem cells provide lower cost and off-the-shelf access that can result in a broader appeal and access.

Mesenchymal stem cells (MSCs) have been characterized by multi-lineage differentiation (adipogenic, osteogenic, and chondrogenic) and by the ability to self renew (5, 6). Additionally, MSCs have been shown to exhibit paracrine or trophic properties of angiogenesis and immunomodulation (7). One of the earliest recognized theories for mechanism of action of cells with regenerative properties was hypothesized by Cohnheim in the late nineteenth century. He believed bone marrow-derived cells could migrate through the bloodstream to distant sites of injury and participate in tissue regeneration (8).

Mesenchymal stem cells are a class of adult stem cells arising from tissues, such as adipose, bone marrow, and many others. It has been demonstrated that MSC frequency in adipose tissue is correlated to blood vessel density (9, 10). MSCs are typically found in a perivascular environment and have recently been termed pericytes (10, 11). These pericytes are released and activated by injury signals. Initial investigations suggested that MSCs functioned by a simple lineage differentiation into various terminal tissue types, but their function has been proven to be a much more complex adaptive set of modes of action (7, 11). These activated MSCs take their instructions from the microenvironment of cells and cytokines produced in the injury site and, therefore, can provide an injury-specific or adaptive response (10). This adaptive ability of MSCs makes them unique in the realm of therapies as their therapeutic responses may be able to adapt to the real needs of the patient depending upon the specific injury/disease process.

Mesenchymal stem cells can be sourced from most tissues with blood vessels. However, adipose is perhaps a more desirable source due to abundance and rapid expansion rate in culture (7, 12, 13). Initial research and commercialization in veterinary regenerative medicine was focused on use in equine orthopedics, given the high monetary value of performance horses (14–17). Stem cell therapy has also been applied in canine orthopedics (18–22). The paracrine mechanisms of action of stem cells described above make them a therapeutic tool to reduce the pain and inflammation of OA with a potentially extended duration of effect.

This paper presents the results of a prospective, randomized, masked, placebo-controlled clinical study, designed to evaluate a target dose of a novel allogeneic, adipose-derived, MSC preparation for safety and efficacy in client-owned dogs with OA of one or two joints. Considering the previously discussed MSC information, the hypothesis is that allogeneic stem cells can provide a therapeutic benefit in canine OA. The presentation of this clinical study is a necessary contribution to the field as it provides allogeneic MSC therapy data that have not been previously published. The paper describes efficacy evaluated from both investigator and dog-owner perspectives in a single study, as well as safety of characterized cells. The results of this study are useful in understanding veterinary use of stem cell therapy in OA, and by extension, may increase understanding of human stem cell therapy.

## Materials and Methods

### Clinical Study Objectives

The primary objective of this study was to confirm that a single dose of allogeneic, adipose-derived MSCs, delivered intraarticularly to either one or two joints, was significantly more effective than a placebo for treatment of OA in dogs. The secondary objective was to provide safety data for this cellular therapy in the target population – dogs with OA. The data were submitted to the Food and Drug Administration (FDA) to support potential regulatory approval of an Investigational New Animal Drug Application.

### Study Design

This was a prospective, randomized, masked, placebo-controlled, multi-site clinical study using client-owned dogs diagnosed with OA. In order to be enrolled, dogs must have had OA that was present in only one or two of the following joints: hips, elbows, stifles, or shoulders. The study length was 60 ± 4 days from treatment to final examination. The MSC product tested, allogeneic adipose stem cells (aASC), is a proprietary formulation of cultured stromal cells derived from adipose tissue harvested from a single disease-screened canine donor.

There were two groups in this study: Group A treated with aASC and Group B treated with placebo (saline). Each group had a target enrollment of 50 dogs. There were nine clinical study sites, all within the United States, with one investigator at each site. The dogs enrolled in this study were client-owned and were maintained in the owner’s residence location by the owners. The dogs were brought to the investigators site for treatment and evaluation at the study defined time points. Institutional Animal Care and Use Committee (IACUC) approval was not required because this study was conducted at private veterinary clinics in the United States to collect data for an Investigational New Animal Drug Application.

### Eligibility

Dogs had to be at least 9 months of age and have a body weight greater than 2.5 kg to be enrolled in the study. They could be male or female. There was no breed restriction. Any physiological status was allowed except pregnant, lactating, or in estrus. Study investigators evaluated the dogs for OA, which could be present in only one or two of the allowed joints: hip, elbow, stifle, or shoulder. Physical examination and radiographs were used to confirm OA diagnosis. The investigators also performed an assessment of pain upon manipulation of the one or two selected joints (Table 1). The score (1, 2, 3, 4, or 5), if only one joint was affected, must have been ≥3 for a dog to qualify for enrollment. If two joints were affected, one must have been scored ≥3 and the combined score must have been ≥5. To be eligible, test results for complete blood count, clinical chemistry, and urinalysis must have been within normal limits or any abnormality must have been considered not clinically relevant in the investigator’s opinion. Overall, except for OA, the dogs must have been in good health based on veterinary physical examination and clinical pathology results, with no known malignant neoplasia or interfering benign neoplasia.

**Table 1:** **Veterinary pain on manipulation scoring system**.

Score	Description of score
1	No response detectable to palpation or manipulation of the limb
2	Mild response detectable to palpation or manipulation of the limb
3	Moderate response detectable to palpation or manipulation, such as turns head toward limb
4	Severe response detectable to palpation or manipulation such as withdraws limb upon minimal movement of joints, vocalizes, or becomes aggressive
5	Does not allow palpation or manipulation

The investigator assessed that there were no health conditions that could interfere with proper evaluation of outcome measures and that the dogs were in compliance with specific diet, medication, and supplements use. Therapies, such as NSAIDS, analgesics, and supplements, were allowed if the dog had been on the treatment for at least 30 days prior to treatment and remained on the treatment at the same dose for the duration of the study. Steroids and injectable joint products were not allowed during the study or within 30 days before treatment. Diet must have been stable for 14 days before treatment and remained the same during the study except for joint-specific diets, which must have been started at least 60 days before treatment. Acupuncture, chiropractic, and laser therapy were not allowed within 20 days of treatment or at any time during the study.

In order for a dog to be enrolled, the owner had to confirm that the dog had persistent pain and/or lameness for at least 3 months prior. In addition, the owner scored the dog on three specific measures, termed the “Client-Specific Outcome Measures (CSOM)” (Table 2). In this evaluation, the owner selected three activities for their dog that were impaired by OA. The scores (0, 1, 2, 3, or 4) for each activity were totaled and the composite score must have been ≥5 for the dog to be enrolled in the study. The owner signed a consent form to participate and agreed to cooperate with study requirements.

**Table 2:** **Client-Specific Outcome Measures (CSOM) scoring system**.

Score	Description of Score
0	No problem
1	Mildly problematic
2	Moderately problematic
3	Severely problematic
4	Impossible

### Treatment Group Assignment and Randomization

Each study site was expected to enroll 10 patients: 5 from Group A and 5 from Group B. Dogs that met all the eligibility requirements were enrolled and allocated randomly to either Group A or B according to a randomization chart for exclusive use at a given study site. For those sites that completed their initial 10 assigned cases and could enroll more cases, additional randomization charts were provided.

### Cell Product Manufacturing/Characterization

The test article, aASC, was manufactured from adipose tissue collected from a single donor dog that was evaluated for exposure to or presence of 26 canine pathogens per agreement with the FDA. This donor dog was a purpose-bred research dog and the collection of the adipose tissue was conducted at a USDA licensed and AAALAC accredited research facility under an approved IACUC protocol by a board-certified veterinary surgeon. The donor dog passed complete clinical examinations before and after the tissue collection process. After the adipose collection procedure, the dog was recovered and monitored for 30 days and then adopted out to a family. This donor dog was not otherwise involved in this clinical study. The cells were grown in tissue culture flasks, using a proprietary media and harvested after passage 4. The product was formulated in a proprietary commercial cryopreservation solution containing 10% dimethylsulfoxide (DMSO) without any serum source. The test article was frozen in a 0.7 mL volume in a cryovial and stored in vapor phase of a liquid nitrogen dewar at the manufacturing site. The cells were shipped in a dry shipper (vapor phase liquid nitrogen) and then stored on-site at the investigator’s clinic in a vapor phase liquid nitrogen dewar.

The aASC cells were manufactured in accordance with rigorous standard operating procedures and lot release criteria. These cells passed testing for sterility and endotoxin and met the identity (CD marker 34−, 45−, MHC Class II−, 44+, and 90+; differentiation positive for adipogenesis, chondrogenesis, and osteogenesis as well as having a stable karyotype) and potency criteria (proprietary VetStem derived test). These criteria are derived from FDA guidelines and a key stem cell industry document that specify the types of criteria to identify adipose MSCs (23).

The target dose of aASC was 12 × 10^6^ viable adipose stem cells in a 0.7 mL volume, which was supplied in cryovials. This target dose was derived from three sources: (1) direct experience of the authors in therapy of more than 5,000 clinical dogs with OA using autologous stem cell therapy (cultured and uncultured); (2) outcome data from aASC therapy of clinical canine OA cases in Australia by Monash University and Australian Veterinary Stem Cells LTD (manuscript submitted); and (3) animal and human adipose stem cell therapy outcomes in the literature. Upon thawing, the aASC product was used directly. The average cell viability upon thawing has been evaluated in laboratory testing to be 85.1% (unpublished data). The placebo (negative) control article was a 0.7 mL volume of saline, and was frozen in single-dose cryovials that appeared identical to test article cryovials.

### Treatment Protocol

Investigators were appropriately trained, qualified veterinarians. They injected affected joints (maximum of 2) of animals in Group A with 0.7 mL of test article aASC in commercial cryopreservation solution and in Group B with 0.7 mL of the control article.

Each dog was appropriately sedated per investigator’s normal protocol to allow complete intraarticular injection of the assigned product. Group B control animals were eligible to receive test article (for the joints under study) in the 2 weeks after they completed the 60-day veterinary exams and owner forms.

Exercise was restricted after intraarticular injection. The owners were instructed to restrict exercise to leashed walking for 5 min twice daily for post-injection days 1–10; leashed walking 10–15 min twice daily for days 11–30; and to wait until after day 30 for longer leashed walks and off-leash exercise.

### Masking Methodology

The investigators and staff members involved with evaluations, as well as the dog’s owner were masked to the identity of treatment group assignments. A label, masking the syringe from the veterinarian performing the injections, hid the identity of the contents of treatment syringes. Only the Sponsor’s study monitor and one investigator site representative had access to the group assignments sheets and only the Sponsor study monitor had access to the treatment group designation. At the end of the treatment and evaluation period for each animal (Day 60 time point), after the last data were collected for a particular animal, the un-blinded investigator site representative was given the information on the group assignment of that animal only, for the purpose of discussing post-study treatment of an animal with test article if they were a control group animal.

The Sponsor representative/study report author was blinded to the treatment group assignment until after final review of each enrolled dog for protocol compliance was completed and final decision was made on which animals were to be removed from the study analysis.

### Outcome Measures

The two categories of outcomes for this study were (1) the effectiveness, as compared to a placebo, in the treatment of OA, and (2) the safety of a single allogeneic intraarticular aASC administration.

#### Efficacy Outcome Variables

The primary outcome variable in this clinical study was the predefined owner assessment of three activities on the CSOM at day 60 (±4) as compared to the CSOM baseline assessment (Day 0). At baseline, the owner selected these measurement activities with guidance from the clinic staff. This is a customized way to evaluate each individual dog’s problems using a standard scoring system rather than using a standard set of activities that may not be relevant to that particular dog. One owner for each dog, rather than multiple owners, provided CSOM evaluations for all time points. The owners indicated how problematic certain activities were, compared to when the dog was normal or did not have OA (Table 2 for scoring system). This indictor has been reported as an accurate measure of drug intervention outcomes in OA (24–26). Each dog was classified as either a treatment success or a treatment failure. Treatment success of an individual dog was defined as a decrease of ≥2 score points in total CSOM score at Day 60 compared to the score at baseline.

The secondary outcome efficacy variables were the veterinary assessment of pain on manipulation (Table 1) and the overall study global outcome score by the owner and by the veterinarian. The global scores were rated as improved, no change, or worsened at Day 60 and success for an individual dog was defined as improved. Treatment success for veterinary pain on manipulation was defined as improvement at Day 60 by at least 1 score point in the total evaluation score from baseline, with no individual score (either joint if two were treated) worsening from baseline.

#### Safety Outcome Measures

This study was designed to evaluate safety based on the veterinary evaluations and owner health observations. The veterinary evaluations included physical examinations and post-injection observations. The owner observations included a diary for recording any abnormal observations of their dog and a report of health that was completed either via telephone (days 15 and 45) or at the veterinary clinic visits (days 0, 30, and 60). Additionally, the owner recorded any change in medications or supplements for their dog. If a veterinary or owner observation was significant, it was reported as an adverse event (AE) and investigated by the attending veterinarian and the Sponsor veterinarian. In addition, investigators were asked to evaluate the AEs and whether or not, in their opinion, the AE was related to treatment. AEs are defined by the FDA and are categorized as serious or not serious. Serious, according to the following definition in FDA regulations at 21 CFR 514.3, means *an adverse event that is fatal, or life-threatening, or requires professional intervention, or causes an abortion, or stillbirth, or infertility, or congenital anomaly, or prolonged or permanent disability, or disfigurement*. An AE is not the same as an adverse reaction. An adverse reaction is an AE that is related to, i.e., caused by, the experimental intervention administered.

### Statistical Methods

The individual enrolled dog was the experimental unit, with the initial target of 50 dogs receiving the test article, and 50 receiving the control article. The hypothesis was that the test article injected intraarticularly would provide a successful outcome (dog classified as a treatment success based on the CSOM evaluation) in a significant number of dogs in comparison to the control group. Significantly more treatment successes with the test article versus the control article based on owner assessment would demonstrate that a target dose of test article cells is therapeutic. Differences were deemed statistically significant using two-sided tests at alpha = 0.05.

#### Primary Outcomes

Treatment success based on CSOM scores on Day 60 was statistically evaluated using methods appropriate for binomial data (the GLIMMIX procedure in SAS, SAS Institute, Cary, NC, USA). A binomial distribution was assumed and a logit link used. Treatment group was included in the model as fixed effects. The Kenward-Rogers adjustment of degrees of freedom was employed. Summary statistics included the percent success by treatment.

#### Secondary Outcomes

Treatment success based on (1) the veterinary assessment of clinical outcomes on Day 60 and (2) pain on manipulation on Day 60, and (3) owner assessment of clinical outcomes on Day 60 were each assessed using methods appropriate for binomial data measured once (the GLIMMIX procedure). A binomial distribution was assumed and a logit link used. Treatment group was included in the model as a fixed effect. The Kenward-Rogers adjustment of degrees of freedom was employed. Summary statistics include the percent success and 95% CI by treatment for each of these outcomes.

## Results

### Group Enrollment Distributions

The treatment and control groups were generally evenly distributed with regard to exclusions, joints treated, breed distribution, age, weight, and sex. The number of dogs enrolled and the number of dogs excluded in the study by treatment group are shown in Table 3. The exclusions in this analysis were due to non-compliance with the protocol or to low site numbers. Only major non-compliance issues, such as failure to substantially follow the exercise restriction requirements or non-disclosure of pre-existing health conditions, were grounds for exclusion. Due to an investigator site dropout before treating any dogs, a total of 93 dogs were treated. Eleven dogs were removed due to non-compliance with the protocol. Due to low enrollment at sites 1, 7, and 8 (<2 evaluable cases in either the control or test product groups), these three sites were excluded from the statistical analysis of efficacy but included in the safety assessments. Thus, 74 dogs (36 in the control group and 38 in the test group) were used in the final statistical analysis.

**Table 3:** **Study enrollment for efficacy outcome**.

Enrollment	Treated	Control	Total
Total enrolled in study	47	46	93
Excluded-protocol non-compliance	4	7	11
Excluded-low site enrollment	5	3	8
Total evaluated for efficacy	38	36	74

Exclusion bias is always a risk in a clinical study. To be conservative, all dogs were used in evaluation of the safety analysis. The efficacy-exclusions method used in this study follows FDA guidelines wherein a fully blinded review of each enrolled dog for appropriate compliance with the study protocol is done first. In this exercise, 11 dogs were removed. Non-compliance was administered, if it was determined that the dog did not complete the study or an event occurred that would make it impossible to appropriately evaluate the primary outcome variable, the CSOM. Table 4 lists the non-compliance reason(s) for blinded-review exclusions of dogs. As previously mentioned, additional dogs were removed from efficacy evaluation if they were from any clinical site where there were less than two animals in each treatment group, because statistical analysis could not be performed. These sites, by definition, had to be removed from analysis.

**Table 4:** **Rationale for exclusion of each of the 11 non-compliant dogs**.

Description of non-compliance
Unapproved long-acting analgesic given at time of treatment Non-compliance to exercise restriction and unapproved pain supplements given Inconsistent use of Rimadyl, then stopped during study Tramadol added during study; incomplete CSOM Discontinued joint supplement; incomplete CSOM Incomplete CSOM Developed dermatology case; given antibiotics; change of diet Discontinued joint supplement; GI issue Allergies not stable; medications increased; medications added Previously seen inflammatory nodule reappeared and burst Dropped out due to death in family

To demonstrate the relatively even distribution of the selected joints by treatment group, Table 5 presents the joint(s) injected, including whether one or both joints were injected. Bilateral hip and elbow were by far the most prominent locations treated.

**Table 5:** **Injected joint distribution for dogs successfully completing the study (includes dogs excluded from efficacy population)**.

Joints injected	# Treated	# Control	Total
**Both joints injected**			
Hip	14	14	28
Elbow	15	12	27
Stifle	2	3	5
Elbow + Shoulder	1	1	2
Hip + Elbow	1	1	2
Shoulder	1	0	1
Stifle + Hip	1	0	1
Stifle + Elbow	0	1	1
**One joint injected**			
Elbow	5	4	9
Hip	2	1	3
Stifle	1	1	2
Shoulder	0	1	1
TOTAL	43	39	82

The study included a variety of large and small breeds. Additionally, Table 6 shows the dogs enrolled in the study were reasonably distributed by age, weight, and sex between the treated and control groups.

**Table 6:** **Enrolled dogs distribution of age, weight, and sex**.

Parameter		Treated	Control	Excluded
Age (years)	Mean	7.98	8.59	7.73
	SD	±3.56	±3.53	±3.13
Weight (kg)	Mean	31.43	29.39	28.06
	SD	±10.75	±11.83	±5.23
Sex	# Female	26	20	6
	# Male	17	19	5
Number	Total	43	39	11

### Efficacy Outcome Variables

**Figure 1:**
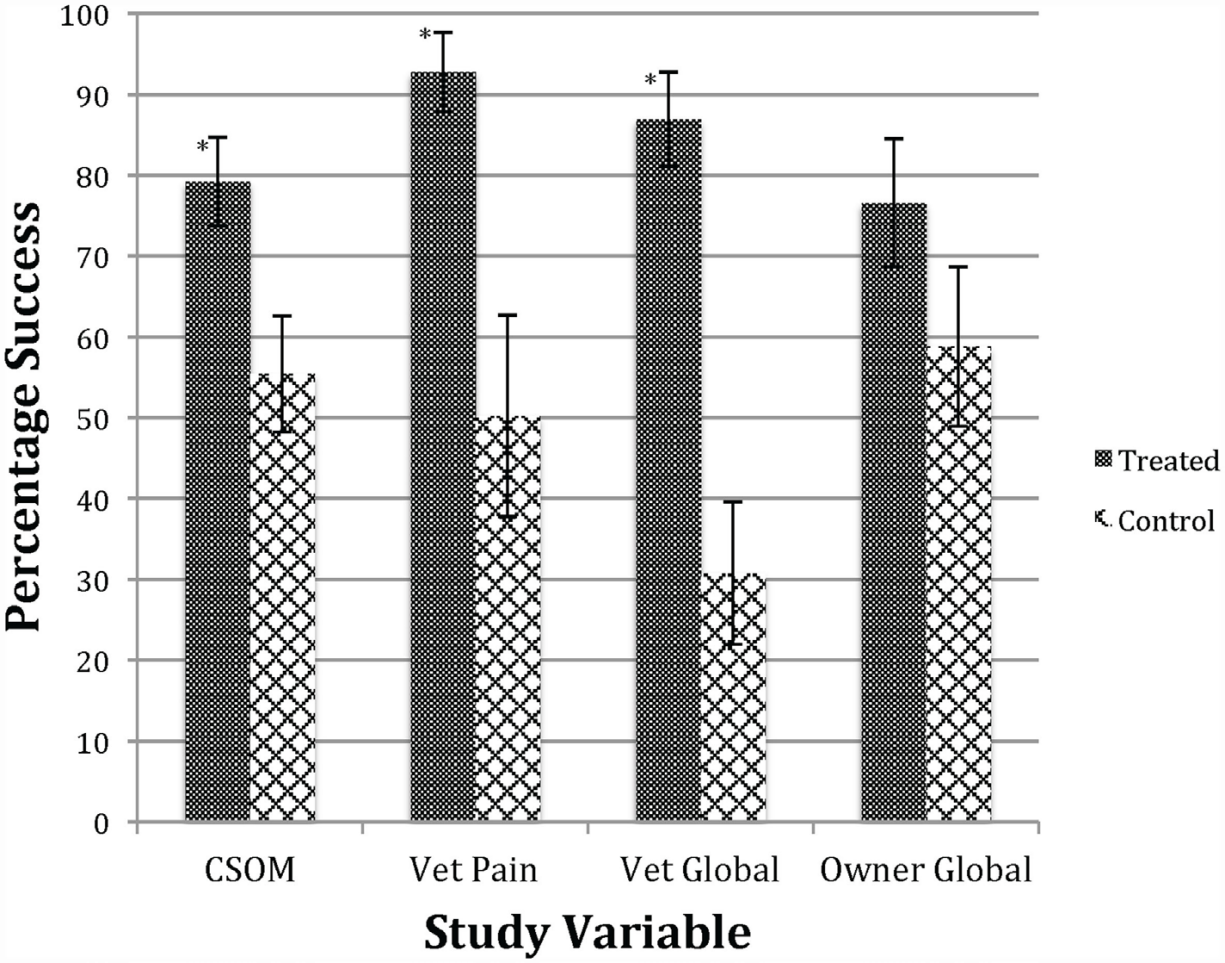
**Success in treatment versus control groups**. *Significant difference *p* < 0.05 between treated and control groups. Bars indicate SE of the mean. This figure shows that the treated group was improved compared to the control group regarding owner CSOM, veterinary assessment of pain, and veterinary global score. See Table 7 for numerical data.

The stem cell treatment was shown to be efficacious compared to placebo, using the primary effectiveness criteria. The summary of the overall outcomes of success versus failure by group with the *P*-value for the statistical evaluation of primary and secondary outcomes, including owner and veterinary clinical assessment measures (global score and pain on manipulation) are shown in Table 7. On analysis, the estimate of the variance associated with the random effects of study site and/or the study site by treatment interaction were negative; site was dropped from the model and the site by treatment interaction variance estimate allowed to be negative (the “nobound” option in GLIMMIX). The data from Table 7 are graphed in Figure 1, which provides a better visual representation of the difference in efficacy between groups. Owner CSOM was statistically improved in the treated versus placebo group (79.21 versus 55.40%), which is an absolute effect size difference of 23.81%. In addition, the two veterinary assessments, veterinary score of pain on manipulation (92.76 versus 50.22%) and the veterinary global score (86.89 versus 30.75%), were also statistically improved in the treatment versus placebo group. The last efficacy measurement, the owner global assessment, improved numerically (76.57 versus 58.81%), but the difference between groups was not statistically significant.

**Table 7:** **Summary of the statistical analysis of the primary and secondary outcome variables success rates**.

Variable	Treated (%)	Control (%)	*P-*value
Owner CSOM (primary)	79.21	55.40	0.0290*
Veterinary pain score	92.76	50.22	0.0170*
Veterinary global score	86.89	30.75	0.0085*
Owner global score	76.57	58.81	0.2128

*Significant difference at p < 0.05.

### Safety Outcome Variables

No significant pattern of differences in observations was seen between the treatment and control dogs in veterinary or owner reports of AEs. For clarity, owners assessed safety every day during the study and data were recorded in a diary. Any significant findings were followed up by the veterinary staff and investigated by the veterinarians during the routine calls and examinations they conducted. The owners were strongly encouraged to record all findings and to call immediately with any adverse health event, so immediate attention could be given to evaluate and treat the dogs, if needed.

In this study, there were a total of 15 AEs reported, 6 in the test group and 9 in the control group. There were two serious AEs reported for each group. In the treated group, the two serious AEs were assessed as not related to, or caused by, the administered cell product: one was a pre-existing degenerative condition (degenerative myelopathy) and the other a respiratory condition acquired/reported 30 days after the end of the study. In the control group, one serious AE was a significant post-injection joint soreness that resolved in 10 days, and the second was facial nerve paralysis that was reported on the last day of the study. The VeDDRA LLT code (Veterinary Dictionary for Drug Related Affairs; Lower Level Term; European Medicines Agency, EMA/CVMP/PhVWP/288284/2007-Rev.8) breakout of reported AEs is shown in Table 8; and there is no pattern of treatment-related AEs.

**Table 8:** **Summary of study adverse events^a^ by disease/system category**.

Category	Treated	Control	Total
Lipoma	1	2	3
Joint pain	0	3	3
Neurological signs	1	1	2
Aggressiveness	2	0	2
Bacterial skin infection	1	0	1
Weight loss	1	0	1
Glaucoma	0	1	1
Respiratory infection	0	1	1
Vomiting	0	1	1
Total	6	9	15

*^a^Includes data from all 93 enrolled dogs*.

Per protocol, any animal in the control group completing the study was offered the option of receiving a stem cell treatment. Of the 46 control animals, 43 opted to receive treatment with the test article after completing the 60-day evaluation period.

## Discussion

In this prospective, randomized, masked, placebo-controlled clinical study, we explored the efficacy and safety of an allogeneic stem cell product in the treatment of OA in dogs. This study was conducted under field clinical conditions in client-owned dogs with OA to test the use of this product in a real-world environment. The cells were produced under controlled laboratory conditions and characterized to demonstrate that the product was a MSC product. Although there is not universal agreement on a single method to identify an MSC, the flow cytometry cluster of differentiation – CD marker results for the five markers, CD34, CD44, CD45, CD90, and MHC II were all within commonly accepted ranges and are commonly reported as standard markers for identification of an MSC (5, 23). The differentiation assays demonstrated the ability of this cell line to differentiate into osteogenic, adipogenic, and chondrogenic lineages.

The efficacy assessments used to evaluate the effects of stem cell therapy on OA in dogs, such as CSOM and pain on manipulation, are those commonly reported in the literature. Veterinary assessment has been the dominant evaluation method historically; however, in recent years, the industry and the FDA have moved to elevate the owner evaluation methods to the primary outcome variable (24, 26). This may be due, in part, to the ability of the owner to assess the dog for longer periods of time and in a more natural and comfortable environment than the veterinary clinic. To the contrary, when a veterinarian is asked to evaluate the dog in an unfamiliar clinical environment, the dog’s natural protection mechanisms and stress reaction can mask the true status of the dog’s clinical signs. The two most common owner assessments have been the Client Specific Outcome Measure (24–27) and the Canine Brief Pain Inventory (28). The CSOM was selected for this study so that the owner, with the veterinarian’s guidance, could most accurately select activities for evaluation that were related to the dog’s specific impairment due to OA. As reference, the FDA does not request force plate or kinetic data as an outcome measure.

The definition of success was applied to each dog and then the percentage of successes was compared between groups, for each assessment tool. The averages for scores were not compared between groups. The FDA requires a more rigorous method of analysis than comparing average scores between groups. Score averaging can buffer extreme variables in the scores of a few animals or the score of a single animal. The assessment of success or failure per each dog is more rigorous than comparing averages of scores, complies with FDA requirements, and has the advantage of reflecting how likely a treatment is to be effective in an individual patient, rather than in a group of patients (29). In this study, the primary variable, the owner CSOM, was statistically improved in the treated versus placebo group during the 60-day study period. In addition, the two veterinary assessments, veterinary assessment of pain on manipulation and the veterinary global assessment, were also statistically improved in the treated versus placebo group. The last efficacy measurement, the owner global assessment, improved numerically, but the difference between the treated and placebo groups did not reach statistical significance.

Global assessment and quality of life measurements are difficult in veterinary medicine, as the assessments are an interpretation through the eyes of the owner and/or veterinarian. There was a significant placebo effect in all measurements, which is commonly reported in veterinary OA studies (30, 31). This placebo effect is often termed the caregiver effect and was recently reported to be an average of 43.1% when veterinarians examined dogs for pain on manipulation of a joint and was 56.9% in an owner-reported lameness questionnaire (30). The placebo effects seen in this study were of similar magnitude. This is precisely the reason for the masked nature of this study. The number of subjects in each group was chosen to detect differences between the treatment and placebo groups, even with a significant placebo effect.

This study design allowed for the investigators to choose dogs for enrollment with OA in one or in two joints. Although it might have been easier to evaluate dogs with only one joint treated, the authors believe that allowing up to two joints more accurately reflects the clinical condition of dogs with OA. As can be seen in the distribution of dogs/joints for this study, 81.7% of the dogs treated were treated in two joints (Table 5).

The cASC product tested in this study was formulated in a 10% DMSO cryoprotectant and injected without washing. The amount of DMSO solution is small (0.07 mL), but one could question whether this might have an anti-inflammatory effect. In a review of PubMed citations, there were no published studies of intraarticular use of DMSO. However, a double-blind, randomized study of 775 human OA patients comparing topical administration of diclofenac against placebo and DMSO carrier was published in 2009. In this study, the products were applied four times daily for 12 weeks and the DMSO product was no more effective than the saline placebo (32).

Safety was also assessed during the 60-day post-injection period in this study. This is the largest randomized, controlled canine stem cell study published and the safety data are important for veterinarians and owners to properly evaluate the risk/benefit of stem cell treatment. The data from this study indicate that there were no significant differences between the treated and control dogs in the safety parameters measured or the AEs reported. In fact, although the numbers are small, there were more AEs reported in the placebo-treated dogs than in the dogs treated with stem cells. To underscore the importance of randomization and blinding, the population of dogs with OA tends to be older and these dogs will have typical age-related issues, such as lipomas, dental disease, ophthalmologic conditions, and many others. Without masking and randomization, it is difficult to assess the meaning of the incidence of these age-related diseases in relation to an experimental treatment. In this study, the groups were not identical, but were comparable regarding the incidence of these types of concomitant conditions. It is important to note that this was a single-dose study. We did not evaluate efficacy or safety of multiple dosing. Repeat dosing, multiples of the target dose, and longer time periods will be evaluated in future safety and efficacy studies and these data will be submitted and reviewed by the FDA in its evaluation of approval for licensing, as with all veterinary drugs.

Why would a clinician consider the use of cell therapy? Therapeutic non-compliance is one reason. The serious problem with owner compliance for administration of chronic drugs, such as NSAIDS, is well known to practitioners. Industry speakers often quote 60–70 days as the average time of administration, even when long-term usage is prescribed. The most common reason cited for not delivering needed medications as prescribed is the owner’s perception that the patient no longer needs treatment, followed by forgetfulness (busy lives), and safety concerns with the drugs (4). NSAIDS are prescribed for daily dose administration, over long periods of time, require prescription refills, and come with label warnings about possible gastrointestinal, renal, and hepatic AEs. Owners are also required to get periodic veterinary examinations and blood work in order to have their prescription refilled. Compounding all these elements makes NSAIDS a class of prescription that incurs naturally poor compliance. As shown in this study and others, stem cell therapy can be effective over a prolonged interval compared to a daily administration drug (18, 19, 21, 22) and has the potential, like other long-acting injectable medications, to address compliance issues and help improve the overall quality and consistency of care for patients with OA. Several studies have shown the use of cell therapy in OA has the possibility of reversing arthritic pathology and even regenerating cartilage (33–35). A stem cell treatment cartilage-regeneration claim was not addressed in this study; such a claim will need to be substantiated by research trials in dogs.

## Conclusion

### Efficacy

The study’s primary objective was achieved and the data confirm that the target dose of allogeneic adipose-derived MSCs delivered intraarticularly to one or two joints was statistically significantly more effective than a placebo in reducing clinical signs of OA in dogs. Both veterinarians and dog owners evaluated stem cell-treated dogs as more often experiencing treatment success than placebo-treated dogs.

### Safety

This single-dose allogeneic stem cell therapy was shown to have no higher incidence of AEs than placebo-treated dogs during the 60-day study period, indicating the potential for stem cells to provide a well-tolerated treatment for OA.

### Overall

Although there are published canine stem cell OA studies, most of these suffer from small sample size, lack of randomization, lack of masking, and/or limited cell characterization data. In addition, many published studies use autologous stem cell sources, whereas this study used a donor-derived allogeneic stem cell source. This is the largest randomized, placebo-controlled canine stem cell study published to date and provides both safety and efficacy data to add to the growing information related to the use of veterinary cell therapy. Stem cell therapy as described in this study has the potential to provide an important canine OA treatment tool for veterinarians: the use of “off-the-shelf” allogenic stem cells without surgical intervention for tissue collection. This large study also provides valuable animal clinical safety and efficacy outcome data to our colleagues developing human stem cell therapy.

## Author Contributions

The authors on this paper qualify as have providing the following overall contributions: (1) substantial contributions to the conception or design of the work; or the acquisition, analysis, or interpretation of data for the work, (2) drafting the work or revising it critically for important intellectual content, (3) final approval of the version to be published, and (4) agreement to be accountable for all aspects of the work in ensuring that questions related to the accuracy or integrity of any part of the work are appropriately investigated and resolved. Specifically, each authors’ contributions were as follows: RH, conception and design, administrative support, provision of study material or patients; data collection, assembly, analysis, and interpretation; manuscript drafting; and final approval of manuscript. KC, JG, SG, SD, KC, MH, TM, PS, and CA all acted as study site investigators and provided study design review, study material or patients, evaluation of individual patients and adverse events, editing/review of manuscript drafts, and final approval of manuscript.

## Conflict of Interest Statement

RH is an employee and shareholder of VetStem Biopharma. The remaining authors declare that the research was conducted in the absence of any commercial or financial relationships that could be construed as a potential conflict of interest.
